# Probing Liquid-Ordered and Disordered Phases in Lipid Model Membranes: A Combined Theoretical and Spectroscopic Study of a Fluorescent Molecular Rotor

**DOI:** 10.1021/acs.jpcb.1c08324

**Published:** 2022-01-10

**Authors:** Gianluca Del Frate, Marina Macchiagodena, Muhammad Jan Akhunzada, Francesca D’Autilia, Andrea Catte, Nicholus Bhattacharjee, Vincenzo Barone, Francesco Cardarelli, Giuseppe Brancato

**Affiliations:** † Scuola Normale Superiore, Piazza dei Cavalieri 7, I-56126 Pisa, Italy; ‡ Center for Nanotechnology Innovation@NEST (CNI@NEST), Piazza San Silvestro 12, I-56127 Pisa, Italy; ¶ Istituto Nazionale di Fisica Nucleare(INFN), Largo Pontecorvo 3, I-56 127 Pisa, Italy; § Consorzio Interuniversitario per lo Sviluppo dei Sistemi a Grande Interfase (CSGI), Via della Lastruccia 3, I-50 019 Sesto Fiorentino, Florence, Italy

## Abstract

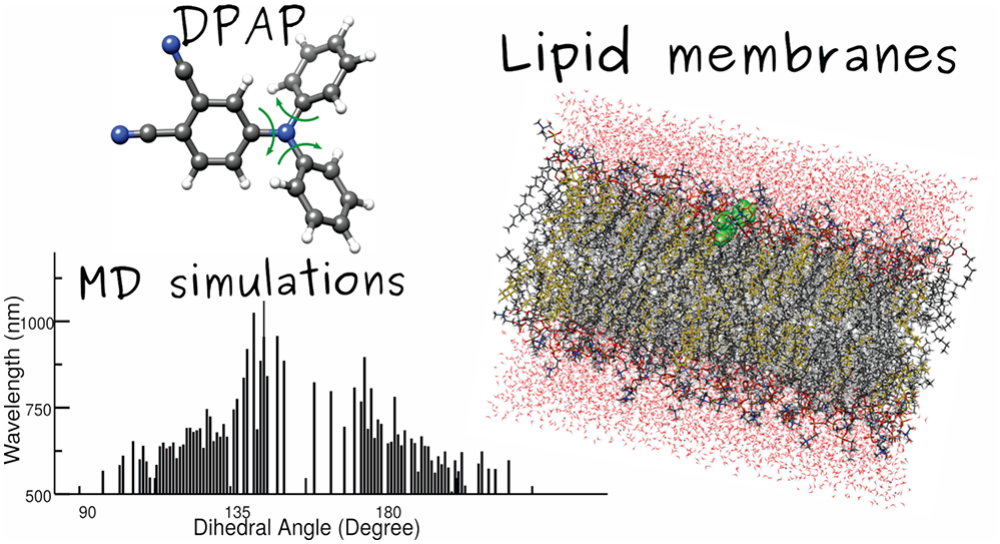

An integrated theoretical/experimental strategy has been applied to the study of environmental effects on the spectroscopic parameters of 4-(diphenylamino)phtalonitrile (DPAP), a fluorescent molecular rotor. The computational part starts from the development of an effective force field for the first excited electronic state of DPAP and proceeds through molecular dynamics simulations in solvents of different polarities toward the evaluation of Stokes shifts by quantum mechanics/molecular mechanics (QM/MM) approaches. The trends of the computed results closely parallel the available experimental results thus giving confidence to the interpretation of new experimental studies of the photophysics of DPAP in lipid bilayers. In this context, results show unambiguously that both flexible dihedral angles and global rotations are significantly retarded in a cholesterol/DPPC lipid matrix with respect to the DOPC matrix, thus confirming the sensitivity of DPAP to probe different environments and, therefore, its applicability as a probe for detecting different structures and levels of plasma membrane organization.

## 1 Introduction

Fluorescent molecular rotors (FMRs) are a class of chemical species capable of modulating their structural and optical properties in response to changes in the viscosity and polarity of the local environment, a feature that makes them particularly suitable for sensing and imaging applications.^1−4^ Such a peculiar capability mainly arises from the intrinsic structural flexibility of the FMRs: typically, this is ascribed to one or more unrestrained and environmentally sensitive dihedral angles, whose internal dynamics largely affects the FMR emission intensity and lifetime upon photoexcitation.^5^ Thanks to these remarkable properties, FMRs can act as viscosity sensors in different environments,^6^ and they have been employed in recent years in order to report on local properties of various biophysical systems.^7−9^ Among others, a very interesting application field concerns the investigation of lipid membrane structures. Indeed, the composition and organization of biological membranes is one of the most relevant topics in molecular biophysics. The modern view identifies a spatially interlaced combination of liquid ordered (L_o_) and liquid disordered (L_d_) phases, enriched, respectively, in saturated and unsaturated lipids, together with different amounts of cholesterol.^10−12^ This nanostructured dynamic assembly of L_d_ and L_o_ phases does not entail definite boundaries but is organized around the cytoskeletal network. Moreover, such a dynamical membrane organization was proposed to be relevant for most membrane processes, such as formation of protein clusters, signal transduction, endocytosis, and cell polarization and motility.^11−15^ In this context, it is not surprising that FMRs have been employed to detect the different phases of cell membranes^16−19^ and to probe the transition from the gel-like to the liquid-crystal phase or, in general, to gain information on the microviscosity of the phospholipid bilayers.^20^ In these studies, it was assumed that more viscous environments may slow down the FMR intramolecular motions,^21^ thus leading to stronger intensities in the corresponding emission spectra and increased fluorescence lifetimes. An effective relation between solvent viscosity (η) and fluorescence quantum yield (ϕ) (or lifetime) is represented by the Forster–Hoffmann model^22^ (i.e., log ϕ ∝ log η), which has been experimentally proved to hold over a wide range of viscosity and polarity scales.^23,24^ It is worth noting that, in turn, lipid membrane viscosity can influence crucial membrane-associated functions, including, for example, passive permeability of hydrophobic molecules, active solute transport, and protein–protein interactions.^25^

**Figure 1:**
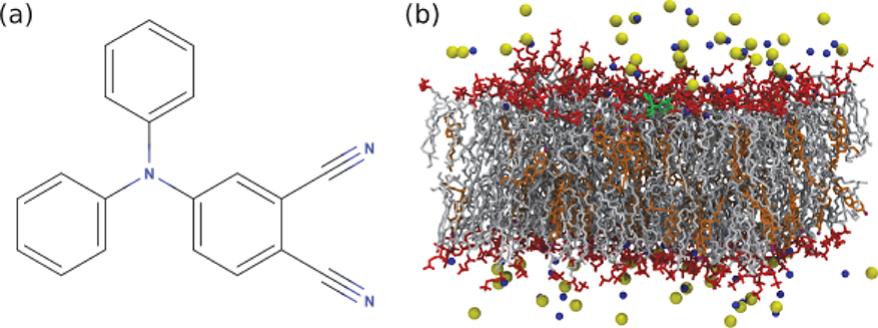
(a) 4-(Diphenylamino)phthalonitrile (DPAP) 2D molecular structure; (b) a configuration of the DPAP rotor (in green) embedded within the DPPC/CHOL matrix. In orange, the cholesterol molecules (with hydroxyl groups in purple); in red and white, DPPC lipid polar heads and nonpolar tails, respectively; in yellow and blue, chloride and sodium ions, respectively. For clarity, water molecules and hydrogen atoms of DPAP and lipids are omitted.

Despite the success of these applications, many features of the complex dynamical organization of the cell membranes still remain elusive. One fruitful approach toward a better understanding of membrane biophysics is to combine fluorescence microscopy and molecular modeling techniques: molecular dynamics (MD), in particular, is commonly adopted for an effective understanding at the atomic level of the dynamics that governs macromolecular functions^26,27^ as well as for investigating basic properties of lipid bilayer models, thus potentially uncovering the subtle interplay between membrane structural rearrangements and lipid dynamics.^28,29^ Several types of FMRs have been reported to date: some of them show a more pronounced dependency on the dielectric properties of the surrounding medium, while others are mainly affected by the molecular free volume of the solvent residues.^30−32^ An optimal combination of strong solvatochromism and viscosity sensitivity was recently shown by the 4-(diphenylamino)phthalonitrile (DPAP, see structure in Figure 1a), whose peculiar *modus operandi* is based upon a barrier-free flexible rotation of its phenyl rings.^33,34^

On these grounds, we combined MD simulations and fluorescence lifetime imaging microscopy (FLIM) to study the application of DPAP as a probe in detecting local order within lipid bilayers representing simple models for both L_o_ and L_d_ phases. To this end, two different phospholipidic systems have been considered, one consisting of pure 1,2-dioleoyl-*sn*-glycero-3-phosphocholine (DOPC) and another one of dipalmitoylphosphatidylcholine (DPPC) enriched with cholesterol (DPPC/CHOL 70:30, Figure 1b). Cholesterol is, in fact, known to increase plasma membrane (PM) viscosity by promoting lipid organization in cellular membranes. Note that the present study was not conceived to provide a proper FLIM calibration toward lipid membranes of variable composition, which necessitates a dedicated study. The use of two limiting L_o_ and L_d_ phase models served mostly the purpose of validating our molecular model, as described in the following. In particular, we adopted a computational strategy that includes the development of a reliable force field (FF) for different electronic states of the molecular probe and its validation through molecular dynamics simulations and spectroscopic calculations in different environments. Noted is that the development of a proper molecular model for this investigation was strictly required since anomalous dye geometries can lead to artifacts in the spectroscopic calculations, and DPAP excitation energies were found to be rather sensitive to its intramolecular configuration.^33,35^ In this work, FF development was tailored toward the DPAP potential energy surface and corresponding gradient in the first excited state as evaluated from quantum mechanical calculations at the time-dependent (TD) DFT level. The sampling of the first electronically excited state (EES) potential energy surface allowed us to effectively simulate excited state properties of DPAP embedded in several solvents (i.e., acetonitrile, cyclohexane, and *o*-xylene) and lipid bilayers and to finally model fluorescence signals as issuing from hundreds of MD trajectory snapshots at a reasonable computational cost. Using MD simulations, it was shown that DPAP rotational dynamics is significantly retarded in more structured (i.e., with high concentrations of cholesterol) lipid bilayers. The obtained results, coming from both computational and experimental investigations, consistently support the use of DPAP as a probe for detecting different structures and levels of plasma membrane organization.

## 2 Materials and Methods

### 2.1 Force Field Parametrization and QM Calculations

The classical force field (intra- and intermolecular terms) used in this work adopts the following energy expression:
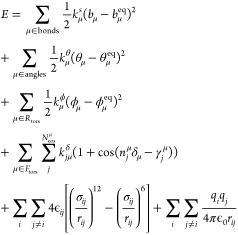
1where *i*, *j* run over atoms and μ runs over the internal coordinates. *R*_tors_ and *F*_tors_ indicate improper and flexible torsions, respectively. Deviations from bonds, angles, and rigid dihedral angles equilibrium values (*b*_μ_^eq^, θ_μ_^eq^, and ϕ_μ_^eq^, respectively) are associated with energy penalties which depend on the corresponding force constants (*k*_μ_^b^, *k*_μ_^θ^, and *k*_μ_^ϕ^). Flexible dihedrals are described by a sum of cosine functions, with *k*_*jμ*_^δ^, *n*_*j*_^μ^, and γ_*j*_^μ^ being the force constant, the multiplicity, and the phase factor of the *j*th cosine. Nonbonded interactions are modeled by using the standard Lennard–Jones and Coulomb potentials.

Force field force constants are analytically computed through the minimization of the Joyce objective function^36^
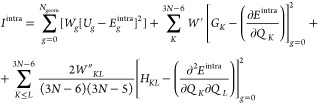
2Here, *K*, *L* run over the normal coordinates, *N*_geom_ is the number of sampled conformations; *U*_*g*_ is the energy difference between the energy of the *g*th conformation and the one computed on the global minimum (*g* = 0). *G*_*K*_ is the energy gradient with respect to the normal coordinate *K*, while *H*_*KL*_ is the Hessian matrix with respect to *K* and *L*. Both *G*_*K*_ and *H*_*KL*_ are evaluated at *g* = 0. The constants *W*, *W*′, and *W*″ weight the several terms at each geometry and can be chosen in order to drive the results depending on the circumstances. The energy, gradient, and Hessian terms, calculated on the obtained equilibrium geometry, are normalized in order to account for the different number of terms and to make the weights independent from the number of atoms in the molecule. The first term of eq 2 is evaluated only if flexible dihedral angles are intended to be parametrized: in this case, *N*_geom_ corresponds to the number of scanned geometries submitted to partial QM optimization. Such process is evaluated under the Frozen Internal Rotation Approximation (FIRA), which assumes that no relevant geometry rearrangements are experienced by the molecule during the scan, except for the scanned dihedral itself. Equilibrium values of eq 1 are simply measured on the minimum geometry. Atomic charges have been computed on the minimum of the reference molecule using the Charge Model 5,^37^ while Lennard–Jones parameters have been taken from the OPLS-AA^38^ force field. Minimum geometry has been located with the time dependent extension of the density functional theory (TD-DFT), using the CAM-B3LYP functional and the SNSD basis set.^39,40^ Environment effects have been included in the geometry optimization procedure by means of the conductor-like polarizable continuum model (C-PCM),^41^ using the butanoic acid (dielectric constant ϵ of 2.9931) as solvent in order to reproduce the specific low dielectric medium of the phospholipidic membranes. Acetonitrile also has been considered in some cases (*vide infra*) to verify the influence of a more polar environment in the computation of chemical properties of interest. Relaxed potential energy surface scan has been performed to accurately parametrize soft dihedral angles, modifying each torsional angle with not uniformly spaced steps (−180°, −150°, −135°, −120°, −90°, −60°, −50°, −30°, 0°, and the positive counterpart). In particular, more points have been considered close to the minima. Vertical electronic transitions have been computed at the CAM-B3LYP/SNSD level using state-specific PCM approaches^42^ to model fluorescence properties. Computations have been performed on 200 molecular configurations extracted from the classical MD trajectories. Single values have been then averaged to obtain the final fluorescence wavelength. All QM calculations were performed with the Gaussian suite of the program (G16).^43^

### 2.2 Simulation Details

The classical MD simulations for DPAP in acetonitrile, cyclohexane, *o*-xylene, and lipid bilayers were performed using GROMACS 4.6.5.^44^ The OPLS-AA force field^38^ was chosen for modeling the *o*-xylene and acetonitrile solvents. In the case of cyclohexane instead, the general amber force field (GAFF)^45^ was used because of its better reproduction of the cyclohexane density experimental value with respect to OPLS-AA. The DOPC bilayer was hydrated with TIP3P^46,47^ water molecules and modeled by means of the CHARMM force field.^48^

To simulate DPAP in a DOPC bilayer, a DPAP molecule was manually inserted, using VMD software,^49^ into a previously equilibrated lipid system containing 200 DOPC lipids and solvated with 5791 water molecules.^35^ The lipid system was originally built up using the CHARMM-GUI membrane builder tool. We carried out an energy minimization of the initial system configuration using the steepest descent algorithm, and then we performed an equilibration for a few nanoseconds in a NpT ensemble, before carrying out the NVT production run (see details below). The final rectangular box size was 8.27 nm, 8.27 nm, and 6.27 nm.

An all-atom DPPC/CHOL bilayer was taken from a previously well equilibrated DPPC/CHOL system,^50^ which contained 1200 lipids with CHOL in a molar concentration of 30%. The system was coarse grained (CG) for 40 μs at 298 K and 1 atm using the CG MARTINI force field for lipids.^51^ The 1200 lipids CG system was initially reduced to 600 lipids with 23.7% molar concentration of cholesterol to perform all-atom MD simulation. This well equilibrated system was then further reduced in the current study to perform all-atoms MD simulation with the CHARMM36 force field.^52^ The final system was comprised of 240 DPPC and 75 cholesterol molecules, evenly distributed between the upper and lower layers, and 1 DPAP molecule was manually inserted into one layer using VMD software.^49^ A 0.15 M NaCl salt concentration was added, and the whole system was solvated with TIP3P^46^ water molecules, with approximately 38 water molecules per lipid. For the DPAP/DPPC/CHOL system, the final box edges were 9.28 nm, 9.15 nm, and 7.22 nm.

Note that initial configurations were minimized by using the steepest descent algorithm implemented in GROMACS using an energy threshold of 10 kJ mol^–1^. Equilibration runs were performed in the isothermal–isobaric ensemble: the systems were heated to 300 K using the velocity rescale method^53^ and the Berendsen barostat^54^ (using coupling constants of 0.1 and 1 ps, respectively) for 500 ps with a time step of 0.2 fs. Production runs were performed in the NVT ensemble: starting from the last configurations of the previous equilibration run, the integration step was increased to 2 fs and the total simulation time was set to about 130 ns for solvent systems and about 230 ns for the lipid bilayers. LINCS^55^ was introduced to fix the fastest degrees of freedom at their equilibrium values. In the case of cyclohexane and DPPC/CHOL lipid, only bonds with hydrogen atoms were kept rigid. Nonbonded interactions were truncated at 1.4 nm. Long-range electrostatic interactions were modeled by means of the particle mesh Ewald (PME) technique^56^ with a spline interpolation of the order 4. System coordinates were stored every 500 steps.

Autocorrelation functions (*C*_p_(*t*)) were calculated as
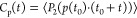
3where *P*_2_ is the second order Legendre polynomial, and *p* is the vector defined as the cross product of the *ij* and *jk* vectors (being *i*, *j*, and *k* three different atoms of the considered molecular structure). Finally, membrane thickness, area per lipid, and deuterium order parameters were calculated using the membrane analysis tool MEMBPLUGIN.^57^

### 2.3 Liposome Preparation

The lipid DOPC (1,2-dioleoyl-*sn*-glycero-3-phosphocholine) and DPPC (1,2-dipalmitoyl-*sn*-glycero-3-phosphocholine) (10 mg/mL in chloroform) were purchased from Avanti Polar Lipids (Alabaster, AL). Cholesterol and low gelling temperature agarose, BioReagent, for molecular biology, were purchased from Sigma-Aldrich (St. Louis, MO). Liposomes of DOPC and DPPC/cholesterol 70:30 were prepared using the standard method.^58^ As an intermediate step for liposome preparation, a thin film of lipid was obtained by evaporating 100 μL of chloroform solution containing 1 mg of DOPC or DPPC/CHOL by placing the sample in a centrifugal evaporator under vacuum for 2 h. The lipid film was hydrated by adding 250 μL of PBS at pH 7.45 at room temperature (DOPC) and at 50 °C (DPPC/CHOL). The vesicles were frozen in liquid nitrogen and then thawed at 50 °C in a water bath. The freeze–thaw cycle was repeated five times. To control liposome size, we performed extrusion using a filter with 0.8 μm pore size. DPAP was solubilized in DMSO and added in liposome solutions. Agarose gel was used to immobilize liposomes as described in ref (59). Agarose was dissolved in PBS at a concentration of 1% w/v. Liposomes were mixed in gel while the agarose was in the fluid state. After mixing, the solution was placed on a glass bottom Petri dish and was left at room temperature for jellification.

### 2.4 Fluorescence imaging and lifetime measurements

Fluorescence imaging and lifetime measurements were performed by means of a Leica TCS SP5 SMD inverted confocal microscope (Leica Microsystems AG) equipped with an external pulsed diode laser for excitation at 405 and 470 nm and a TCSPC acquisition card (PicoHarp 300, PicoQuant) connected to internal spectral detectors. Laser repetition rate was set to 40 Hz. The image size was 256 × 256 pixels, and the scan speed was usually set to 400 Hz (lines per second). The pinhole aperture was set to 1.0 Airy. Samples were imaged using a 100× 1.5 NA oil immersion objective (Leica Microsystems). Emission was monitored in the 480–525 nm and 540–580 nm ranges using the built-in acousto-optical beam splitter detection system of the confocal microscope. Acquisitions lasting until about 100–200 photons per pixel were collected at a photon counting rate of 100–500 kHz.

## 3 Results and Discussion

### 3.1 Excited-State Structure and FF Development

**Figure 2:**
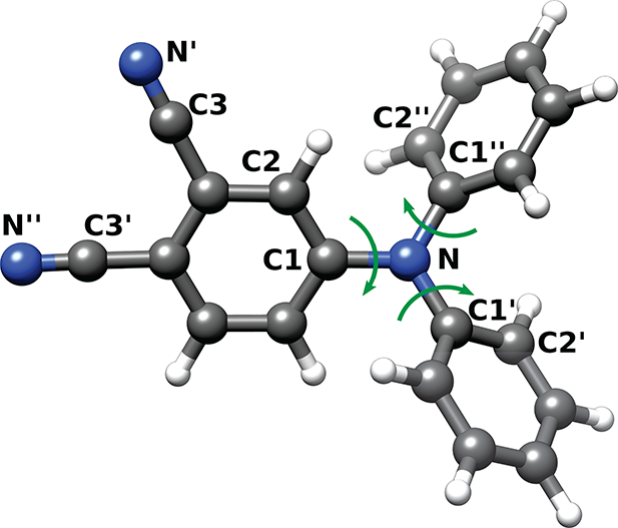
DPAP propeller-like conformation as optimized in butanoic acid (solvent effect modeled by the C-PCM). Flexible dihedral angles are indicated with green arrows. The *ipso* (C1, C1′, and C″), the *ortho* (C2, C2′, and C2″) carbon atoms are labeled in black together with the nitrogen (N′ and N″) and the carbon (C3 and C3′) atoms of the two cyano groups.

The optimized excited state structure of the DPAP molecular rotor (shown in Figure 2) adopts a propellerlike shape in order to minimize steric hindrance among the three phenyl rings, with the central moiety defined by the three *ipso* carbon atoms and the aminic nitrogen adopting a nearly planar conformation.

**Figure 3:**
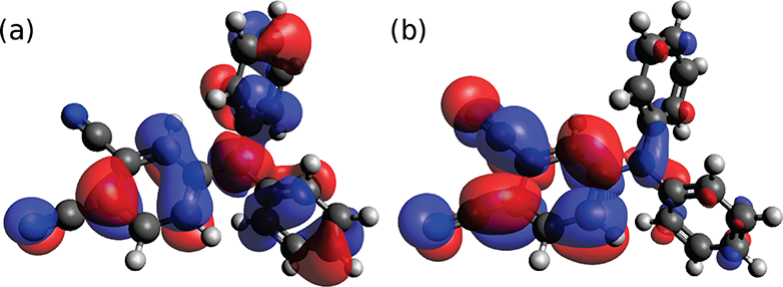
Frontier molecular orbitals of DPAP: HOMO (a) and LUMO (b). An isosurface value of 0.02 au has been used.

As a preliminary investigation, we performed a comparison between the internal coordinates which significantly change when going from the GS to the EES structure (see Table 1). This kind of analysis is important, since the differences between the GS and EES geometries are generally associated with the Stokes shifts in the UV–vis spectrum.^60^ Significant structural rearrangements are observed for the chemical bonds involving the cyano groups. From the analysis of the DPAP HOMO and LUMO (graphically shown in Figure 3), it is apparent that these alterations can be ascribed to the migration of the electronic density from the unsubstituted phenyl rings to the dicyano substituted moiety, taking place upon the excitation process. It is worth noting also the change in the *ipso* region geometry, with a widening of  and a narrowing of  and  angles in the EES with respect to the GS. These alterations can be due to the higher electronic delocalization which takes place in the EES, owing to the inductive and resonance effects on the unsubstituted phenyl rings due to the dicyano aromatic moiety.^61,62^ To better highlight the electronic rearrangement upon electronic excitation, we computed the charge transfer (CT) index^63^ for both ground and first-excited states. The extent of the electronic rearrangement is defined as the distance between the two centers of the density increment and depletion regions, respectively, upon electronic excitation (graphical representation of the two charge density centers (i.e., positive and negative) is depicted in Figure S2 in the Supporting Information). In cyclohexane, the computed CT length is 2.173 Å and the charge is 0.62 e.

**Table 1:** Comparison between Ground State (GS) and Electronically Excited State (EES) DPAP Geometry Optimized^a^

geometric parameter	GS	EES
C3′–N″ (Å)	1.156	1.165
C3–N′ (Å)	1.155	1.164
N–C1 (Å)	1.382	1.406
N–C1″ (Å)	1.430	1.406
N–C1′ (Å)	1.430	1.396
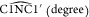	121.16	118.96
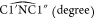	117.55	121.52
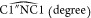	121.30	119.51
C2C1NC1″ (degree)	20.73	45.18
C1″NC1′C2′ (degree)	54.36	33.16
C2″C1″NC1′ (degree)	–125.00	–142.62
C1C1″C1′N (degree)	–0.22	–0.41

a Atom labeling in Figure 2.

As mentioned in section 2, the FF atomic point charges have been computed on the minimum of the reference molecule using the Charge Model 5. To verify the influence of the specific solvent on the DPAP atomic charges, the same population analysis has been performed also using acetonitrile (ϵ = 35.688) as the solvent. Negligible differences on the estimated charges were found, with the largest deviation being about 0.023 *e*. Such small differences in the atomic charges slightly affect the dipole moment value, going from 21.19 D in butanoic acid to 23.14 D in acetonitrile. Overall, the observed insensitivity of the estimated atomic charges values to the surrounding medium allows for the employment of the same set of charges (the one computed in butanoic acid, in the present case) for all the investigated environments in the following MD simulations.

A comparison between the GS and EES atomic charges computed in acetonitrile is shown in Table S1 of the Supporting Information. The choice of acetonitrile for this analysis allows for an effective comparison with the GS force field, which was developed by considering this environment during the previous parametrization.^35^ The main differences have been observed for the cyano nitrogen atoms (N′ and N″, according to Figure 2), which become more negative after the electronic excitation, and for N and C1 atoms (7.15 × 10^–2^ and 4.71 × 10^–2^*e*, respectively). In this case, the lower atomic charge values confirm a more pronounced electronic density in the EES, as already indicated by the interpretation of the geometric parameter alteration. The high degree of intramolecular charge transfer is reflected also by the important difference between the GS and the EES dipole moments: in acetonitrile, the calculated dipole moment is 12.79 D for the GS and 23.14 D in the EES.

**Figure 4:**
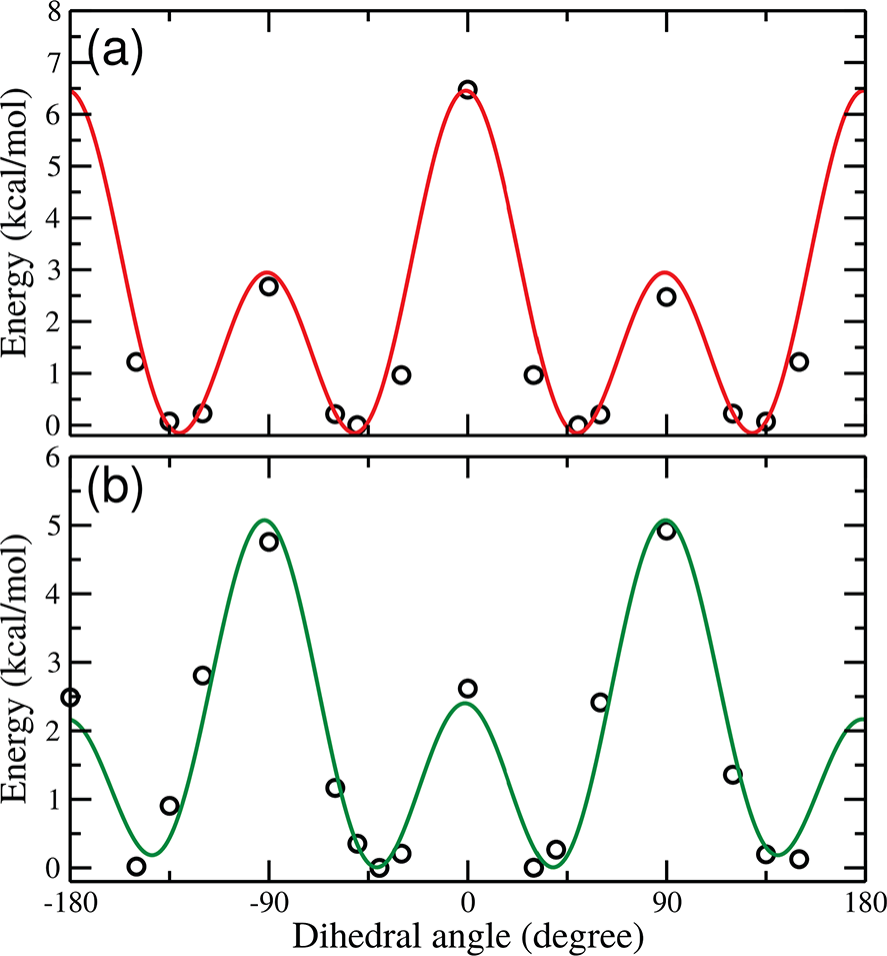
Potential energy profiles of dihedral angle **1** (a) and **2**/**3** (b). QM data, open circles; MM data, solid lines.

The bonded terms of the molecular FF have been derived according to the protocol described in section 2.1. A fundamental step in this procedure is represented by the parametrization of the flexible dihedral angles. The DPAP conformation is mainly affected by three dihedral angles, which define the ring torsions with respect to the central amine group: dihedral **1** (C2C1NC1″, see Figure 2 for labeling), dihedral **2** (C1″NC1′C2′) and dihedral **3** (C2″C1″NC1′). The last two dihedral angles (**2** and **3**) are equivalent. The related DFT potential energy profiles have been used to derive the torsional potential terms of the DPAP excited-state FF. The result of the fitting procedure is shown in Figure 4, where the MM potential energy ruling the dihedral angle **1** (Figure 4a) and **2**/**3** (Figure 4b) are compared with the corresponding DFT reference data.

Dihedral angle **1** shows four symmetry-related energy minima at ±130° and ±50°. Two energy barriers (i.e., ∼7 kcal/mol and ∼3 kcal/mol) rule the interconversion among these conformers, corresponding to the planar and orthogonal geometry of the considered ring with respect to the central amine moiety. The situation is reversed for dihedral angles **2**/**3**, with the orthogonal conformer being energetically disfavored. In such a case, the four energy minima are located at about ±45° and ±135°. Moreover, in both the energy panels of Figure 4, a satisfactory agreement between the optimized classical FF and the DFT reference data can be observed, thus allowing a reliable sampling of the first EES potential energy surface of the DPAP molecular rotor by means of the new FF. The whole parameter set is available in the Supporting Information (Tables S2–S7).

As a further analysis, the potential energy profiles of the scanned flexible torsions can be compared with the corresponding ones obtained for the ground electronic state case.^35^ All the energy barriers are higher for the EES for both the investigated torsions and their relative height is exchanged: in the GS, the highest energy barrier for dihedral **1** corresponds to the orthogonal configuration, whereas it is the planar conformation in the EES. A reversed situation is observed for dihedral **2**/**3**. This remarkable trend is due to the electron delocalization which involves the two unsubstituted rings in the EES, which leads, in turn, to an extra stabilization energy when dihedral **2**/**3** is nearly planar.

### 3.2 Model Validation in Organic Solvents

The FF developed in the previous section has been tested and validated by means of MD simulations of DPAP in acetonitrile, *o*-xylene, and cyclohexane. The different solvation shells experienced by the analyzed FMR in its first excited state have been described by means of the radial distribution function (RDF) computed between the dye and the solvent molecules center of mass (COM). The obtained profiles, shown in Figure S3, point out well-defined first solvation shells for cyclohexane and *o*-xylene. In the former solvent, a first peak of height 1.75 is found at 6 Å of COM distance, and a second peak with comparable height (1.25) at 9 Å. In the case of *o*-xylene, the first peak has a height of ≈1.5 at 5 Å; the second peak is not well resolved and it spans a large area from 8 to 12 Å. Only a few molecules of acetonitrile instead are able to closely approach the solute, since the height of the RDF first peak is significantly lower if compared to the previous ones. On the contrary, the second solvation shell is easy to detect from the distribution profile being located at approximately 8 Å with a height of 1.1.

The distributions of the three flexible dihedral angles, evaluated in all the MD simulations, confirm the reliability of the first-excited state FF for the DPAP molecule. Indeed, as it is shown in Figure S4 for the case of acetonitrile, each dihedral angle selectively populates the four corresponding energy minima. High energy conformations are avoided, according to the DFT energy profile depicted in Figure 4, which was the target of the FF parametrization. Each flexible torsion undergoes complete rotations, thus being able to properly populate the four energy minima predicted by the QM calculations. However, the time required for a complete rotation strongly depends on the environment. In particular, it is well-known that the viscosity of the solvent affects the solute internal dynamics, with more viscous media decelerating the rotation of dihedral angles involving large chemical moieties. Regarding our system, a qualitative picture can be easily gained by monitoring the dihedral angle evolution during the sampled simulation time. Inspection of Figure S5a shows that in acetonitrile (the less viscous solvent considered) 1 ns is enough to observe oscillations of the considered dihedral (dihedral angle **1**, in the present case) from 120° to 60° and vice versa. On the other hand, even after 5 ns unequivocal large amplitude oscillations were not yet detected in the more viscous solvents cyclohexane and *o*-xylene (Figure S5b,c), for which similar viscosities are reported in the literature.^64,65^

Focusing on the acetonitrile (ACN) case, where a higher flexibility of the DPAP internal dynamics is allowed, we noted that the torsional angles rotate simultaneously in order to decrease the steric hindrance between the aromatic rings, this meaning that the three torsions are highly coupled. This phenomenon was already pointed out in the previous work on DPAP ground state and can be better appreciated by looking at Figure S6: the first rotation takes place after almost 1 ns of simulation and the following at roughly 1.4 and 2 ns. It is noteworthy that in the time interval (5 ns) considered in this figure only small oscillations are allowed for dihedral **1**, which is ruled by a low energy barrier of ≈3 kcal/mol. The first 5 ns of simulated time are, instead, not sufficient to overcome the energy barrier of 7 kcal/mol ruling the dihedral angle **1**. Finally, all the energy minima of dihedral **2**/**3** (separated by barriers of 2.5 and 5.0 kcal/mol) are populated. From a quantitative point of view, we further confirmed the observed trend among the three considered solvents by computing along the entire MD trajectories the time autocorrelation function (ACF) of the rotation of both ring 1 torsional angle (τ_rot_^dih^) and of the whole molecule (τ_rot_). In the latter case, the axis perpendicular to the plane defined by the three *ipso* carbon atoms **C1**, **C1′**, and **C1″** was considered as the reference vector. The calculated quantities, collected in Table 2, indicates that the internal and external flexibility of DPAP decreases in the order ACN > cyclohexane > *o*-xylene.

**Table 2:** Dynamic Properties of DPAP in Various Solvents

solvent	τ_rot_ (ps)	τ_rot_^dih^ (ps)	τ_fl_ (ns)^33^
acetonitrile	8.17 ± 0.02	7.98 ± 0.02	2.61
cyclohexane	89 ± 3	94 ± 5	9.16
*o*-xylene	117 ± 16	126 ± 13	12.5

This result is in line with the different solvation shells sampled during the MD simulations, already seen in Figure S3: in the first solvation shell *o*-xylene molecules are closer to the solute center of mass than cyclohexane molecules, thus obstructing to a larger extent the DPAP internal and global movements. Moreover, from inspection of Table 2, a clear correlation between τ_rot_ (or τ_rot_^dih^) and the experimental fluorescence lifetime τ_fl_ emerges, thus corroborating the fact that more viscous and hindering environments promote radiative processes, as a consequence of the obstruction to the rotation of the dye subgroup adopting the twisted intramolecular charge-transfer (TICT) state. This remarkable finding (graphically shown in Figure S7), which relates an experimental quantity with a computational prediction, could lead to interesting implications, considering that FMRs are often used within highly viscous media such as silica-based nanoparticles, in order to increase fluorescence lifetimes and to be fruitfully used for imaging applications.^66^

DPAP emission spectra have been experimentally determined in recent years,^33^ proving that the fluorescence (in contrast to the absorption, which is insensitive to the environment) is highly solvatochromic since it shows a red-shift of up to 120 nm going from the less polar cyclohexane (having a ϵ of 2.016) to the most polar solvent acetonitrile. According to our procedure (explained in section 2.1), TD-DFT calculations were performed on dye configuration frames extracted each 250 ps of simulations, for a total of 200 fluorescence energy calculations for each of the considered environment. It has to be recalled that the considered structures correspond to S_1_ configurations, so that the computed emissions occur from the first excited state S_1_. The frontier molecular orbitals have been already shown in Figure 3. During the aforementioned computations, solvent coordinates were not considered, and environment effects have been modeled through the PCM scheme. The CAM-B3LYP functional was chosen because of its reliability with chemical systems involving charge-transfer upon excitation, and it was already successfully applied in a previous study.^35,67^

The obtained results are summarized in Table 3. It is apparent from our theoretical calculations that the solvent polarity has a significant impact on the overall spectroscopic outcome: the higher the dielectric constant of the medium (i.e., ϵ), the higher is the emission energy.

**Table 3:** Experimental and Theoretical Maximum Emission Peak Wavelength (nm) and Stokes Shift of DPAP in Different Environments^a^

	ACN	cyclohexane	*o*-xylene	DOPC	DPPC/CHOL
	(35.69)	(2.02)	(2.54)	(2.99)	(2.99)
	Fluorescence				
exptl (nm)	552	431	471		
calcd (nm)	682 (±127)	442 (±29)	470 (±34)	496 (±31)	503 (±38)
	Stokes Shift				
exptl (nm)	231	107	144		
calcd (nm)	353 (±147)	117 (±44)	145 (±51)	173 (±43)	

a In parentheses is the dielectric constant of the solvent. Stokes shift values are computed by considering the absorption peak wavelength determined in our previous work.^35^ Experimental data are not available for DOPC and DPPC/CHOL bilayers.

In the low-polarity cyclohexane solvent the theoretical prediction of the DPAP emission wavelength (442 nm) is close to the corresponding experimental quantity (431 nm). Considering the results of our previous work (where an absorption peak at 325 nm was found), we can also estimate the Stokes shift provided by the CAM-B3LYP/SNSD/PCM model, leading to a value of 117 nm, in fair agreement with the experimental one (107 nm). In the case of *o*-xylene, the experimental value of the fluorescence energy is reproduced with high accuracy, with an underestimation of only 1 nm. Taking into account the already computed value for the absorption, the estimated Stokes shift (144 nm) differs from the experiment by only 1 nm.

Finally, the emission wavelength computed for acetonitrile (the highest polarity solvent) is 682 nm, which overestimates the experimental value by more than 100 nm. Also the Stokes shift is overestimated, suggesting that the polarity effect in this case is amplified. However, beside that, it is worth noting that the polarity-sensitivity of DPAP is correctly described at the CAM-B3LYP/SNSD/PCM level, thus showing that this model is able to reproduce the experimental trend. This statement can be easy confirmed even if the related statistical errors reported in Table 3 are taken into account.

**Figure 5:**
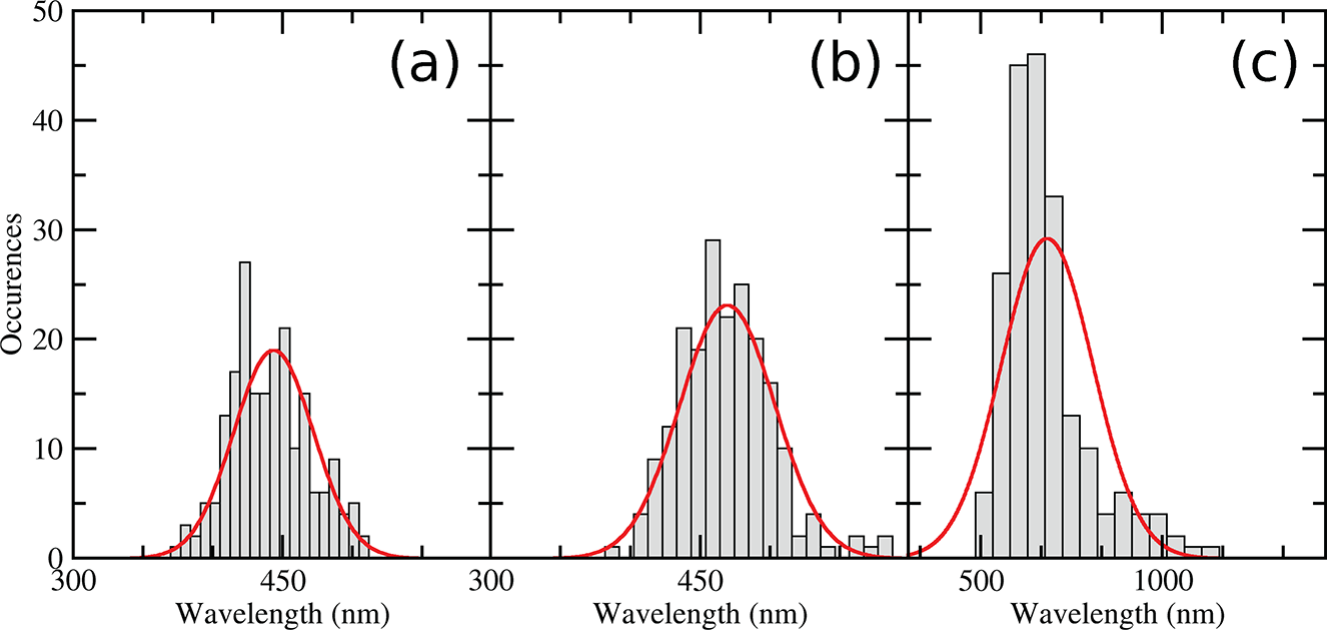
Distribution of the emission wavelengths computed for 200 DPAP geometries extracted from the MD trajectories in cyclohexane (a), *o*-xylene (b), and acetonitrile (c). The solid lines represent the fitting to a Gaussian curve. For sake of clarity a different number of bins have been used in the three panels.

The larger errors observed for ACN with respect to the other solvents can be rationalized by noting that the corresponding RDF profile is somewhat less structured, as discussed in section 3.2. This may be due to a poor description of the solute–solvent interactions, which were not addressed in the present study. The lack of a defined solvation shell in ACN results into a larger internal flexibility, thus increasing the conformational variability on which the spectroscopic investigations have been performed. On the contrary, in cyclohexane and *o*-xylene, the structured surrounding solvation shells prevent DPAP from large amplitude motions, which can lead to larger deviations of the computed fluorescence energy. This is graphically displayed by the distribution of the computed emission wavelengths for each liquid, as shown in Figure 5.

**Figure 6:**
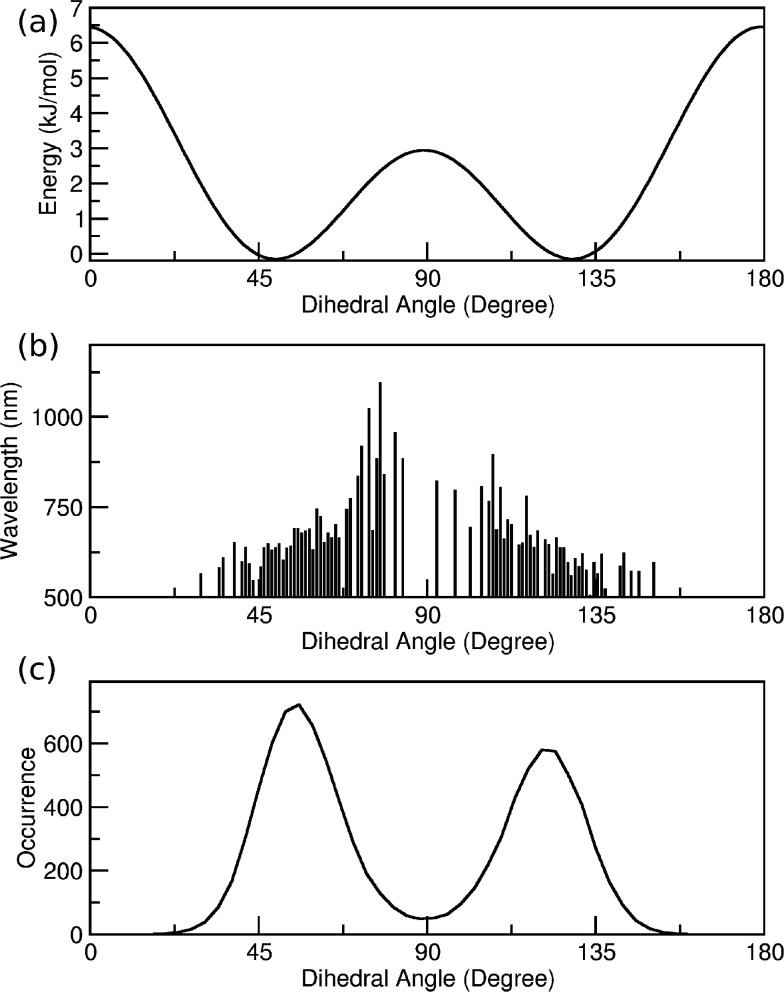
Conformational dependence of the fluorescence wavelength computed in acetonitrile. (a) Energy profile of dihedral angle **1**. (b) Relation between DPAP emission energy and dihedral angle **1** amplitude. (c) Dihedral **1** distribution for the selected conformations taken from the MD trajectory and used for fluorescence wavelength calculations.

Moreover, looking at the acetonitrile case, an apparent effect of the conformational changes on the predicted optical property arises from inspection of Figure 6, where the value of dihedral angle **1** is related to the corresponding emission wavelength. Higher values in the emission energy are associated with the considered torsional angle within the interval 70–120° (Figure 6b) while lower values arise from torsional angle values close to the corresponding energy minima (as indicated by the panel a in Figure 6 where the potential energy curve is shown). In Figure 6c, the dihedral **1** distribution of the 200 conformations extracted from MD trajectory used for the fluorescence wavelength calculation is shown: as expected, the dihedral angle **1** correctly populates the related energy minima.

### 3.3 Probing Lipid Phase in Model Bilayers through DPAP Fluorescence Lifetime

**Figure 7:**
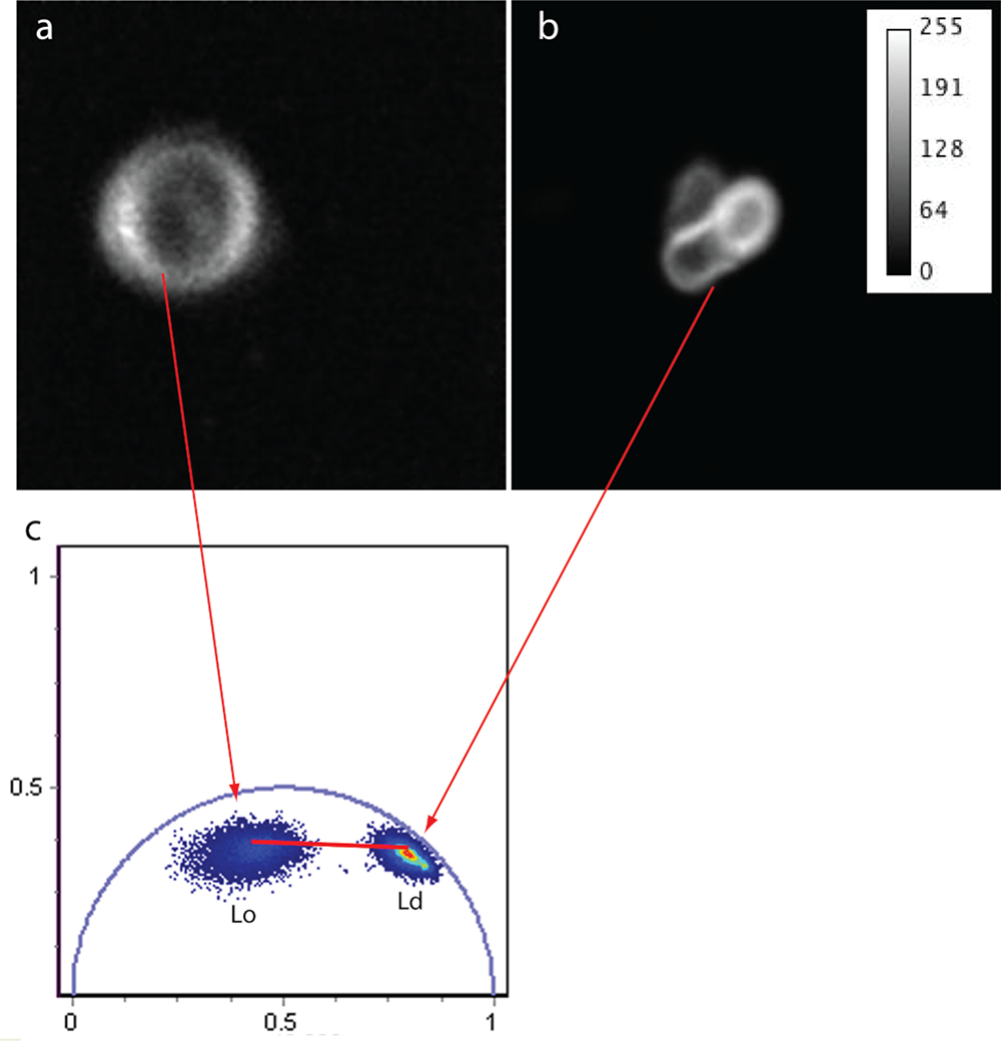
ph-FLIM of multilamellar vesicles characterized by homogeneous lipid phases. (a) Fluorescence image of a multilamellar vesicle characterized by the homogeneous L_o_ phase (i.e., DPPC/cholesterol). (b) Fluorescence image of a multilamellar vesicle characterized by the homogeneous L_d_ phase (i.e., DOPC). (c) Phasor plot relevant to vesicles (a and b), as superimposed on the same diagram: the segment connecting the averages of the two reference phasor clouds is depicted in red.

The sensitivity of DPAP photophysics to local viscosity, as observed in previous studies,^33,68^ prompted us to test such a FMR for probing lipid membrane structure. To this end, we set up two liposome solutions of pure DOPC and DPPC–cholesterol (70:30), as described in the Materials and Methods section, which are known to provide convenient L_d_ and L_o_ membrane phase models, respectively. Once provided to the liposome solutions, DPAP was readily embedded in the lipid membranes, owing to its highly hydrophobic character that makes it highly insoluble in aqueous solution.^33^ As usual in FMR imaging applications, we focused specifically on the fluorescence lifetime of the dye, since other optical features of the recorded spectra, such as emission intensities and wavelengths, are strongly dependent on local concentration or less sensitive to the microviscosity of the environment. In particular, in order to probe the nature of the lipid phase through DPAP emission lifetime, we adopted the phasor approach^69^ to confocal fluorescence lifetime imaging microscopy (ph-FLIM) as a convenient means to spatially map the phase order in lipid bilayers. Besides, this is a propedeutic step toward probing local order in living cells. The phasor analysis is represented in a polar 2D plot (phasor plot^70^) the cosine (g_*i*,*j*_) and sine (s_*i*,*j*_) Fourier transforms of the normalized emission decay collected in each pixel *i*, *j* of an image. For monoexponential decays, the phasor (g_*i*,*j*_, s_*i*,*j*_) lies on a semicircle (universal circle) of radius 1/2 and center (1/2,0); for multiexponential decays, the phasor lies inside the semicircle. Experimentally, we applied confocal ph-FLIM to DPAP embedded on multilamellar vesicles characterized by homogeneous L_d_ (i.e., DOPC) or L_o_ phases (i.e., DPPC-Cholesterol). Notably, L_d_ or L_o_ phases were found to be characterized by well-distinguishable phasors localized in the phasor plot (Figure 7). The dispersed nature of the phasor “cloud” owes to the finite precision of our measurements (more collected photons lead to better defined and more compact clouds) and the vesicle heterogeneity (this has a minor effect in this study since we produced liposomes with controlled composition). As expected for its longer lifetime (τ_fl_ = 6.75 ns), the phasor cloud of the more rigid L_o_ phase mapped closer to the (0,0) point as compared to the L_d_ phase (τ_fl_ = 1.93 ns). On the phasor plot, the combinations of distinguishable photophysical states, such as those determined by L_d_ and L_o_ phases, follow a vectorial addition rule, regardless of the number of exponentials.^69−71^ Therefore, we may hypothesize that interleaved L_d_ and L_o_ phases, such as those expected in the plasma membrane of the cell, would fall along the segment that connects the two “reference” phasors. Accordingly, by assuming there is a correlation between lipid composition and structural order, ph-FLIM applied to DPAP could, in principle, help in determining the composition of the membrane with the submicrometer resolution typical of confocal microscopy.

### 3.4 DPAP Molecular Dynamics in Different Lipid Phases

The influence of lipid membrane viscosity on DPAP dynamics was further investigated by means of MD simulations. A DPAP molecule was manually embedded into pre-equilibrated planar DOPC and DPPC/CHOL model bilayers, thus reproducing the experimental setup (see the Materials and Methods section for details), and MD simulations of both systems were carried out for about 200 ns after equilibration. In particular, both systems showed structural features of the membrane in agreement with previous MD simulations: for DPAP/DOPC, we obtained an area per lipid of 68.5 Å^2^ and a membrane thickness of 38.1 Å, while for DPAP/DPPC/CHOL the area per lipid (DPPC) was 60.0 Å^2^ and the membrane thickness was 40.0 Å. Once embedded within the bilayer in one of the leaflet, DPAP remained in the hydrophobic region under the lipid headgroup surface, as shown by the density distributions displayed in Figure S8. In DPPC/CHOL, DPAP partitioned between the lipid phosphate groups and cholesterol (i.e., at about the level of the cholesterol hydroxyl groups). Besides, to also better assess the lateral distribution of the FMR within the membrane bilayers, we analyzed the radial distribution functions between DPAP and either the phosphate (i.e., P atom) or cholesterol hydroxyl groups (i.e., O atom), selecting only the lipids belonging to the upper leaflet where DPAP was embedded. As shown in Figure S9, DOPC and DPPC lipids appeared similarly structured around DPAP with a peak centered at about 0.9 nm, while the cholesterol distribution featured a broader distribution peaked at 1.5 nm.

**Figure 8:**
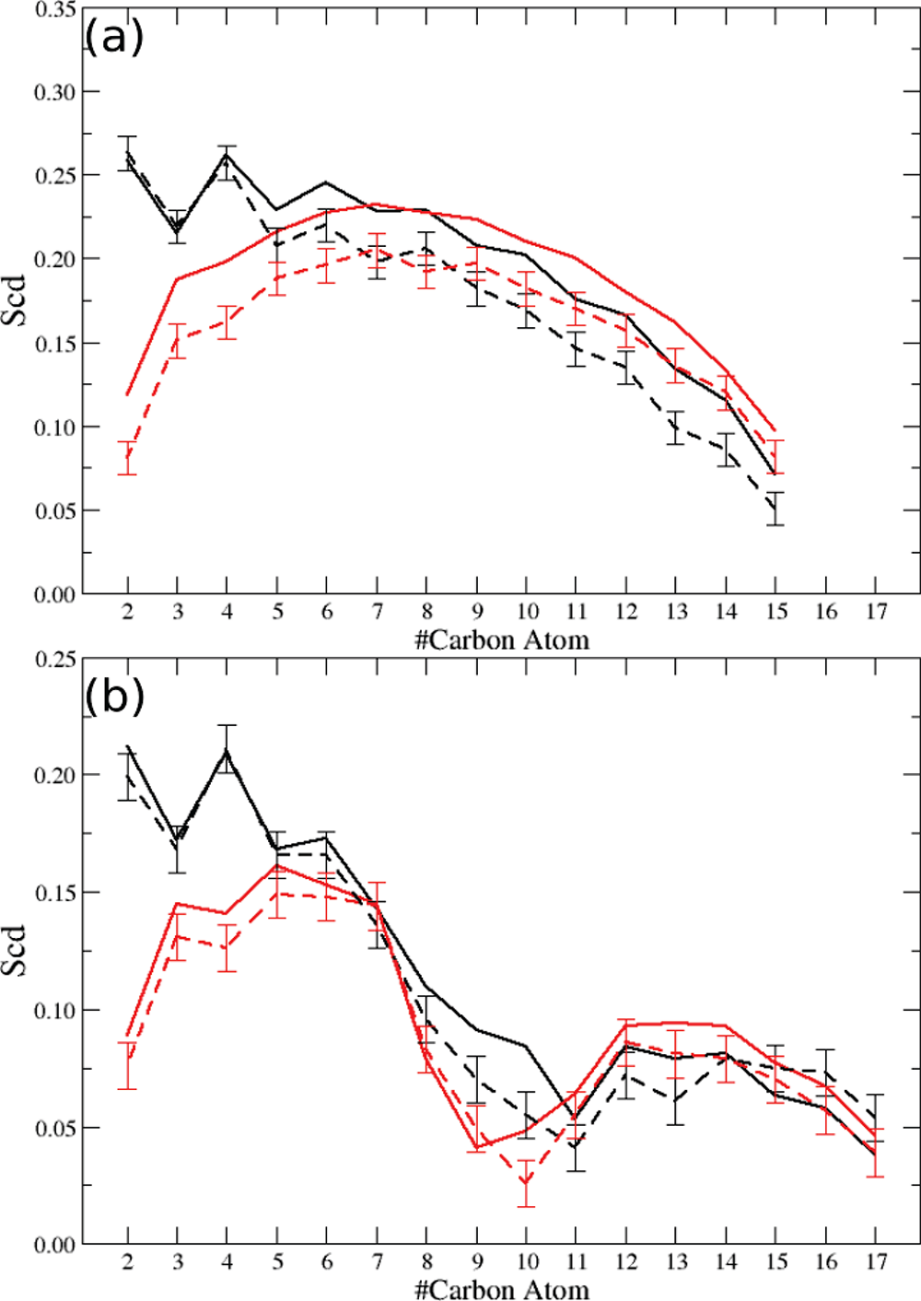
Computed deuterium order parameters: (a) DPPC and (b) DOPC. Solid line, average over all DPPC/DOPC lipids; dotted line, average over DPPC/DOPC lipids within 5 Å from DPAP. In black *sn*-1 chain, in red *sn*-2 chain.

Furthermore, we analyzed the structural effect due to the presence of DPAP within both the DOPC and DPPC/CHOL membrane models by evaluating the deuterium order parameter of the lipids. In particular, in both systems, we considered either the full set of lipids (i.e., DOPC or DPPC) or only the lipids in proximity to DPAP (within a distance of 5 Å from the FMR). The order parameters for each carbon atom of the two lipid alkyl chains (i.e., *sn*-1 and *sn*-2, respectively) were evaluated to provide some information about the lipid structural order within the bilayer. Results are reported in Figure 8. From Figure 8, it can be observed that the order parameters of both bulk DOPC and DPPC lipids nicely agree with results from previous studies^28,72−74^ and show the more ordered structure of the latter with respect to the former, as due also to the condensing effect of cholesterol. When considering lipids in contact with DPAP, we noticed only slight deviations of the order parameters with respect to both bulk lipids, within the statistical noise. These findings highlight that DPAP does not significantly perturb the underlying lipid bilayer structure, a desirable feature for a molecular probe.

In Table 4, the computed rotational relaxation times of both the ring 1 torsional angle (τ_rot_^dih^) and of the whole molecule (τ_rot_) in DOPC and DPPC/CHOL bilayer are reported. First, we observed that DPAP rotational dynamics recorded in the membrane systems was greatly retarded if compared to the one measured in solution, owing to the enhanced viscosity of the lipid matrix environment. In DOPC bilayer, both relaxation times were about ∼10 ns (Table 4). In the presence of cholesterol and within a more ordered lipid phase (i.e., DPPC/CHOL), the phenyl ring rotational dynamics slowed down to ∼25 ns and global rotations (τ_rot_) were further retarded to about 44 ns. Interestingly, considering DPAP fluorescence lifetimes (DOPC, τ_fl_ = 1.93 ns; DPPC/CHOL, τ_fl_ = 6.75 ns) we also noticed a comparable trend, thus confirming that, within a similar chemical environment (i.e., lipid matrix), local viscosity effectively hinders rotational motions and, in turn, induces longer emission lifetimes. This is particularly relevant in view of microscopic techniques aiming at imaging subcellular compartments through the fluorescence lifetime, a convenient concentration-independent optical property.

**Table 4:** Dynamic Properties of DPAP in DOPC and DPPC/CHOL Bilayers^a^

environment	τ_rot_ (ns)	τ_rot_^dih^ (ns)	τ_fl_ (ns)
DOPC	12 (2)	12 (2)	1.93
DPPC/CHOL	44 (10)	25 (5)	6.75

a Errors are in parentheses.

Furthermore, DPAP emission was evaluated computationally in DOPC and DPPC/CHOL bilayers. The maximum emission peak wavelength of DPAP in DOPC and DPPC/CHOL were located at 496(±31) and 503(±38) nm, respectively. Therefore, the predicted emission wavelength was similar for the two phospholipidic bilayers, the small discrepancy being ascribable to the different DPAP configurations sampled during the classical MD simulations. Moreover, the obtained emission values fall into the wavelength ranges considered for the evaluation of the fluorescence lifetime (see section 2.4), thus confirming from an experimental point of view the reliability of the results issued from our model.

## 4 Conclusions

In this work, we analyzed the FMR DPAP as a molecular probe for detecting ordered (L_o_) and disordered (L_d_) phases in plasma membranes. For this purpose, we used a combined experimental and computational approach, which can be easily extended to study complex phenomena occurring upon interaction between lipids and molecular probes. In the first part, a molecular model of DPAP was developed and validated. A purposely tailored classical force field for modeling the first electronic excited state of DPAP was obtained using TD-DFT calculations as reference data. Three different solvents (acetonitrile, cyclohexane, and *o*-xylene) were considered to assess the force field through MD simulations of DPAP in solution while in its excited-state. We observed that the rotor internal and global dynamics is affected by the viscosity of the environment, besides other specific solute–solvent interactions. The corresponding fluorescence spectra, as computed by means of TD-DFT calculations on hundreds of uncorrelated MD snapshots, reproduced the solvatochromic trend fairly well and the Stokes shift of the emission signals.

In the second part, DPAP was investigated when embedded within two different lipid bilayers, namely, pure DOPC and DPPC with cholesterol, as membrane models for the L_d_ and L_o_ phases, respectively. Using confocal fluorescence lifetime imaging microscopy, we obtained a significantly different optical response providing a τ_fl_ of about 2 and 7 ns for DOPC and DPPC/CHOL, respectively. MD simulations of DPAP within the same membrane systems revealed that both internal and global rotations of the probe were significantly retarded with respect to the tested solutions, particularly in the L_o_ phase model. Notably, results showed a consistent correlation between the fluorescence lifetime and rotational dynamics within the lipid matrix systems under consideration. Therefore, our work highlights, once more, the sensitivity of DPAP toward its microenvironment and suggests its use as a probe for the detection of lipid structures as those characterizing plasma membrane organization.

## Supporting Information Available

DPAP atomic charges obtained in acetonitrile for the GS and EES (Table S1); EES DPAP FF (Tables S2–S7); radial distribution functions between DPAP and solvents center of mass (Figure S3); DPAP dihedral distribution function of the three dihedral angles in acetonitrile (Figure S4); DPAP time dependent dihedral distribution function for dihedral angle 1 for the first 5 ns of simulation in solvents (Figure S5); DPAP time dependent dihedral distribution function for the three dihedral angles for the first 5 ns of simulation in acetonitrile (Figure S6); correlation between DPAP rotational correlation time and experimental fluorescence lifetimes in the considered environments (Figure S7); density profile along the *Z*-axis for the DPPC/DOPC phosphorus atoms, CHOL oxygen atoms, and DPAP (Figure S8); and radial distribution functions between DPAP and DPPC/DOPC phosphorus atoms and CHOL oxygen atoms (Figure S9) (PDF)
