# Supplementary material for: Probing Liquid-Ordered and Disordered Phases in Lipid Model Membranes: A Combined Theoretical and Spectroscopic Study of a Fluorescent Molecular Rotor
Source: J Phys Chem B. 2022 Jan 10;126(2):480–91. doi: 10.1021/acs.jpcb.1c08324 (PMC8785181; doi:10.1021/acs.jpcb.1c08324)
Supplement: Supplementary file 1 — jp1c08324_si_001.pdf [file jp1c08324_si_001.pdf]

Supporting Information for "Probing liquid-ordered and disordered phases in lipid model membranes: a combined theoretical and spectroscopic study of a fluorescent molecular rotor"

Gianluca Del Frate,[†] Marina Macchiagodena,[†,‖] Muhammad Jan Akhunzada,[†] Francesca D'Autilia,[‡] Andrea Catte,[†] Nicholus Bhattacharjee,[†] Vincenzo Barone,[†,¶,§] Francesco Cardarelli,[†] and Giuseppe Brancato*,[†,¶,§]

†*Scuola Normale Superiore, Piazza dei Cavalieri 7, I-56126 Pisa, Italy*

‡*Center for Nanotechnology Innovation@NEST (CNI@NEST), Piazza San Silvestro 12, I-56127 Pisa, Italy*

¶*Istituto Nazionale di Fisica Nucleare(INFN), Largo Pontecorvo 3, I-56127 Pisa, Italy*

§*Consorzio Interuniversitario per lo Sviluppo dei Sistemi a Grande Interfase (CSGI), Via della Lastruccia 3, I-50019 Sesto Fiorentino (FI), Italy*

‖*Present Address: Dipartimento di Chimica "Ugo Schiff", Universitá degli Studi di Firenze, Via della Lastruccia 3, I-50019 Sesto Fiorentino, Italy*

E-mail: giuseppe.brancato@sns.it

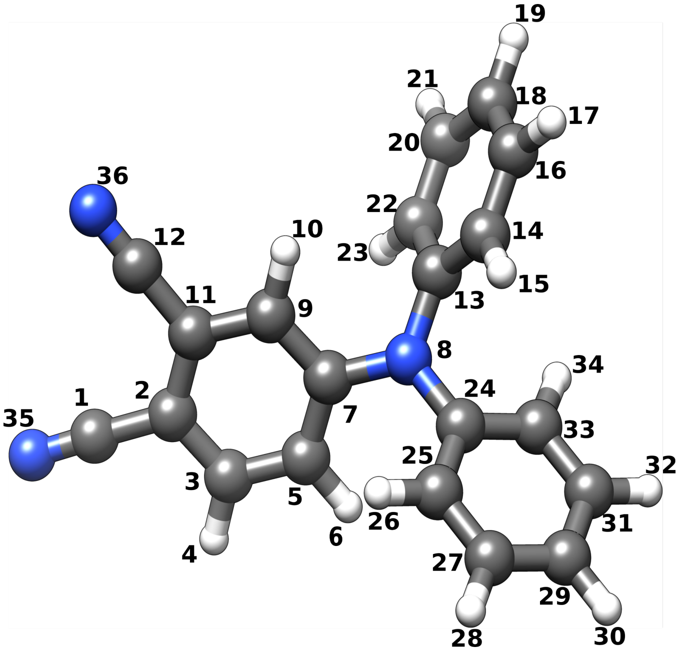

Figure S1: DPAP atom labelling.

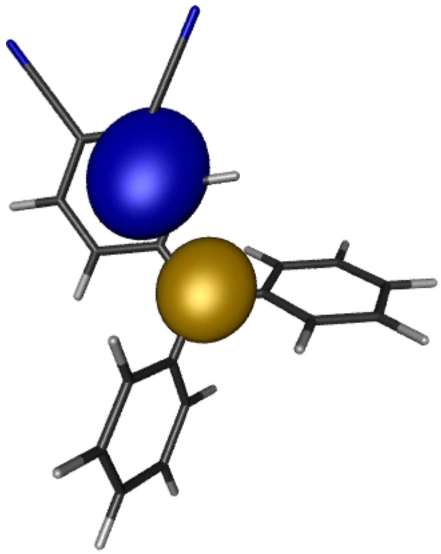

Figure S2: Graphical representation of the charge density centroids (blue: positive, yellow: negative) of DPAP in cyclohexane, as issuing from B3LYP/SNSD calculations (details are described in the main text).

Table S1: Comparison between GS and EES atomic charges (e) of DPAP in acetonitrile, issuing from CAM-B3LYP/SNSD calculations using the Charge Model 5. For atom labeling see Figure S1.

Atom	GS	EES
1 C	0.194	0.165
2 C	0.001	-0.028
3 C	-0.064	-0.083
4 H	0.130	0.122
5 C	-0.082	-0.121
6 H	0.121	0.109
7 C	0.137	0.090
8 N	-0.295	-0.224
9 C	-0.074	-0.067
10 H	0.123	0.128
11 C	0.022	-0.033
12 C	0.207	0.163
13 C	0.100	0.118
14 C	-0.093	-0.077
15 H	0.115	0.125
16 C	-0.096	-0.085
17 H	0.112	0.123
18 C	-0.101	-0.075
19 H	0.110	0.122
20 C	-0.096	-0.087
21 H	0.112	0.123
22 C	-0.095	-0.074
23 H	0.114	0.125
24 C	0.100	0.122
25 C	-0.093	-0.075
26 H	0.116	0.121
27 C	-0.096	-0.087
28 H	0.112	0.123
29 C	-0.101	-0.072
30 H	0.110	0.123
31 C	-0.096	-0.086
32 H	0.112	0.123
33 C	-0.094	-0.074
34 H	0.114	0.125
35 N	-0.400	-0.450
36 N	-0.383	-0.454

Table S2: DPAP force field non-bonded parameters. For atom labeling Figure S1.

Non-bonded Parameters

atom	σ (nm)	ϵ (kJ/mol)	charge
1	3.65000e-01	6.27600e-01	0.186654
2	3.55000e-01	2.92880e-01	0.019087
3	3.55000e-01	2.92880e-01	-0.059224
4	2.42000e-01	1.25520e-01	0.125066
5	3.55000e-01	2.92880e-01	-0.062729
6	2.42000e-01	1.25520e-01	0.126168
7	3.55000e-01	2.92880e-01	0.124887
8	3.30000e-01	7.11280e-01	-0.282292
9	3.55000e-01	2.92880e-01	-0.062916
10	2.42000e-01	1.25520e-01	0.127709
11	3.55000e-01	2.92880e-01	0.031833
12	3.65000e-01	6.27600e-01	0.192961
13	3.55000e-01	2.92880e-01	0.099550
14	3.55000e-01	2.92880e-01	-0.097983
15	2.42000e-01	1.25520e-01	0.106604
16	3.55000e-01	2.92880e-01	-0.092697
17	2.42000e-01	1.25520e-01	0.104930
18	3.55000e-01	2.92880e-01	-0.104520
19	2.42000e-01	1.25520e-01	0.100278
20	3.55000e-01	2.92880e-01	-0.093007
21	2.42000e-01	1.25520e-01	0.105063
22	3.55000e-01	2.92880e-01	-0.102183
23	2.42000e-01	1.25520e-01	0.104397
24	3.55000e-01	2.92880e-01	0.102670
25	3.55000e-01	2.92880e-01	-0.107760
26	2.42000e-01	1.25520e-01	0.102222
27	3.55000e-01	2.92880e-01	-0.095023
28	2.42000e-01	1.25520e-01	0.104000
29	3.55000e-01	2.92880e-01	-0.110641
30	2.42000e-01	1.25520e-01	0.097695
31	3.55000e-01	2.92880e-01	-0.094128
32	2.42000e-01	1.25520e-01	0.104120
33	3.55000e-01	2.92880e-01	-0.103733
34	2.42000e-01	1.25520e-01	0.104017
35	3.20000e-01	7.11280e-01	-0.356519
36	3.20000e-01	7.11280e-01	-0.344556

Table S3: DPAP force field stretching parameters.For atom labeling Figure S1.

Bonds

ai	aj	r_{eq}(nm)	k^s (kJ/mol nm^2)
1	2	0.1413	331022.256
2	3	0.1398	267669.331
3	4	0.1085	341351.000
3	5	0.1379	333590.929
5	6	0.1082	341351.000
5	7	0.1439	288139.658
7	8	0.1416	259638.001
7	9	0.1378	288139.658
9	10	0.1085	341351.000
2	11	0.1453	217365.339
9	11	0.1400	267669.331
11	12	0.1409	331022.256
8	13	0.1404	259638.001
13	14	0.1403	288139.658
14	15	0.1084	341351.000
14	16	0.1384	333590.929
16	17	0.1085	341351.000
16	18	0.1392	333590.929
18	19	0.1085	341351.000
18	20	0.1394	333590.929
20	21	0.1085	341351.000
13	22	0.1403	288139.658
20	22	0.1382	333590.929
22	23	0.1084	341351.000
8	24	0.1391	259638.001
24	25	0.1408	288139.658
25	26	0.1083	341351.000
25	27	0.1385	333590.929
27	28	0.1085	341351.000
27	29	0.1390	333590.929
29	30	0.1085	341351.000
29	31	0.1399	333590.929
31	32	0.1085	341351.000
24	33	0.1409	288139.658
31	33	0.1378	333590.929
33	34	0.1083	341351.000
1	35	0.1162	1101330.351
12	36	0.1163	1101330.351

Table S4: DPAP force field bending parameters.

Angles

ai	aj	ak	θ_{eq}(degr)	k^{θ}(kJ/mol rad^2)
1	2	3	119.35	632.2333
1	2	11	121.16	515.7457
2	1	35	180.00	668.0000
2	3	4	118.34	310.1840
2	3	5	122.15	650.9733
3	2	11	119.49	162.0124
4	3	5	119.51	329.1979
3	5	6	121.62	329.1979
3	5	7	117.76	658.7915
6	5	7	120.47	297.0103
5	7	8	118.15	668.5092
5	7	9	121.50	274.2746
8	7	9	120.31	668.5092
7	8	13	119.19	240.7903
7	8	24	118.73	240.7903
7	9	10	119.76	297.0103
7	9	11	120.94	751.4922
10	9	11	119.30	310.1840
2	11	9	118.13	162.0124
2	11	12	122.29	515.7457
9	11	12	119.59	632.2333
11	12	36	180.00	668.0000
8	13	14	120.33	668.5092

8	13	22	119.53	668.5092
13	8	24	122.07	240.7903
13	14	15	119.80	297.0103
13	14	16	119.56	658.7915
14	13	22	120.10	274.2746
15	14	16	120.60	329.1979
14	16	17	119.52	329.1979
14	16	18	120.36	648.2884
17	16	18	120.12	329.1979
16	18	19	119.98	329.1979
16	18	20	120.03	648.2884
19	18	20	119.99	329.1979
18	20	21	120.14	329.1979
18	20	22	120.34	648.2884
21	20	22	119.52	329.1979
13	22	20	119.60	658.7915
13	22	23	119.56	297.0103
20	22	23	120.82	329.1979
8	24	25	119.20	668.5092
8	24	33	120.92	668.5092
24	25	26	119.50	297.0103
24	25	27	119.58	658.7915
25	24	33	119.82	274.2746
26	25	27	120.87	329.1979
25	27	28	119.45	329.1979
25	27	29	120.41	648.2884
28	27	29	120.14	329.1979

27	29	30	120.04	329.1979
27	29	31	120.08	648.2884
30	29	31	119.87	329.1979
29	31	32	120.02	329.1979
29	31	33	120.39	648.2884
32	31	33	119.58	329.1979
24	33	31	119.70	658.7915
24	33	34	119.76	297.0103
31	33	34	120.50	329.1979

Table S5: DPAP force field improper torsional parameters.

Improper Dihedrals

ai	aj	ak	al	ξ_{eq}	k^{ξ}(kJ/mol)
11	2	3	4	178.3	75.979
11	2	3	5	-1.4	120.114
35	1	2	3	0.1	0.007
1	2	11	9	179.9	85.688
2	3	5	6	177.2	46.509
3	2	11	12	-179.3	85.688
4	3	5	7	-178.0	65.990
3	5	7	9	-1.2	61.628
6	5	7	9	-176.8	47.552
5	7	9	10	-179.3	47.552
8	7	9	11	-177.5	159.749
7	9	11	2	-0.2	32.329
10	9	11	2	179.7	75.979
9	11	12	36	23.3	0.007
8	13	14	16	178.5	195.234
8	13	22	20	-179.2	195.234
13	14	16	17	-178.6	65.990
22	13	14	16	0.7	61.628
14	13	22	23	-179.9	47.552
15	14	16	18	177.9	78.198
14	16	18	20	-0.5	49.866
17	16	18	19	-1.0	25.381
16	18	20	21	179.8	78.198
19	18	20	22	179.2	78.198
18	20	22	13	1.1	42.113
21	20	22	23	-0.3	25.381
33	24	25	26	-179.0	47.552
8	24	33	31	178.3	195.234
24	25	27	28	-178.9	65.990
8	24	25	27	-178.7	195.234
25	24	33	31	1.1	61.628
26	25	27	29	178.3	78.198
25	27	29	30	179.6	78.198
28	27	29	31	179.9	78.198
27	29	31	33	-0.5	49.866
30	29	31	32	-1.1	25.381
29	31	33	34	177.6	78.198
32	31	33	24	-179.1	65.990

Table S6: DPAP force field improper dihedral angles parameters.

Improper Dihedrals

ai	aj	ak	al	ξ_{eq}	k^{ξ}(kJ/mol)
2	1	3	11	0.4	310.779
5	2	4	3	-0.2	282.979
7	3	6	5	-2.8	92.399
9	5	8	7	-1.1	471.574
8	7	13	24	0.1	28.016
11	7	10	9	0.1	301.297
11	2	9	12	0.1	310.779
13	8	14	22	-1.3	471.574
16	13	15	14	1.4	273.029
18	14	17	16	0.6	284.824
20	16	19	18	0.4	284.824
22	18	21	20	-0.0	284.824
23	13	20	22	-0.8	413.250
24	8	25	33	1.6	471.574
27	24	26	25	1.5	273.029
29	25	28	27	0.2	284.824
31	27	30	29	-0.3	284.824
33	29	32	31	-0.6	284.824
34	24	31	33	-1.2	413.250

Table S7: DPAP force field flexible torsional parameters.

Flexible Dihedrals

ai	aj	ak	al	γ	k^{ϕ}(kJ/mol)	n
9	7	8	24	0.00	3.674	2
9	7	8	24	0.00	4.913	4
5	7	8	13	0.00	3.674	2
5	7	8	13	0.00	4.913	4
13	8	24	25	0.00	-0.059	1
13	8	24	25	0.00	-2.916	2
13	8	24	25	0.00	0.304	3
13	8	24	25	0.00	3.612	4
7	8	24	33	0.00	-0.059	1
7	8	24	33	0.00	-2.916	2
7	8	24	33	0.00	0.304	3
7	8	24	33	0.00	3.612	4
24	8	13	22	0.00	-0.059	1
24	8	13	22	0.00	-2.916	2
24	8	13	22	0.00	0.304	3
24	8	13	22	0.00	3.612	4
7	8	13	14	0.00	-0.059	1
7	8	13	14	0.00	-2.916	2
7	8	13	14	0.00	0.304	3
7	8	13	14	0.00	3.612	4

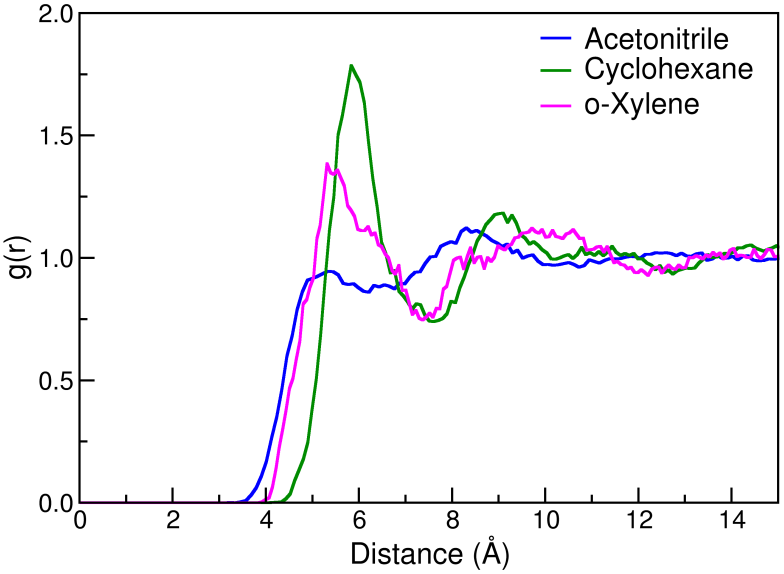

Figure S3: Radial distribution functions between DPAP and acetonitrile (blue), cyclohexane (green) and *o*-xylene (magenta) center of mass.

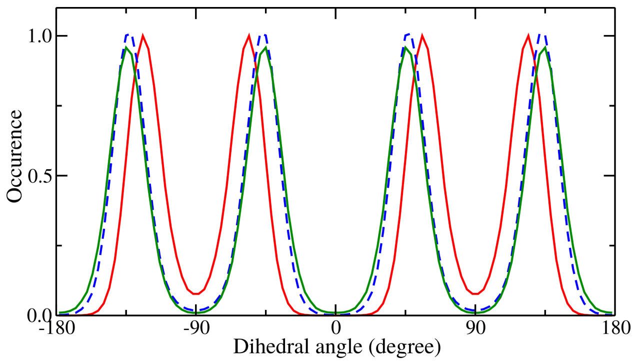

Figure S4: Dihedral distribution function of the three dihedral angles in acetonitrile: in solid red line dihedral angle **1**, in blue dashed line dihedral angle **2** and in solid green line dihedral angle **3**.

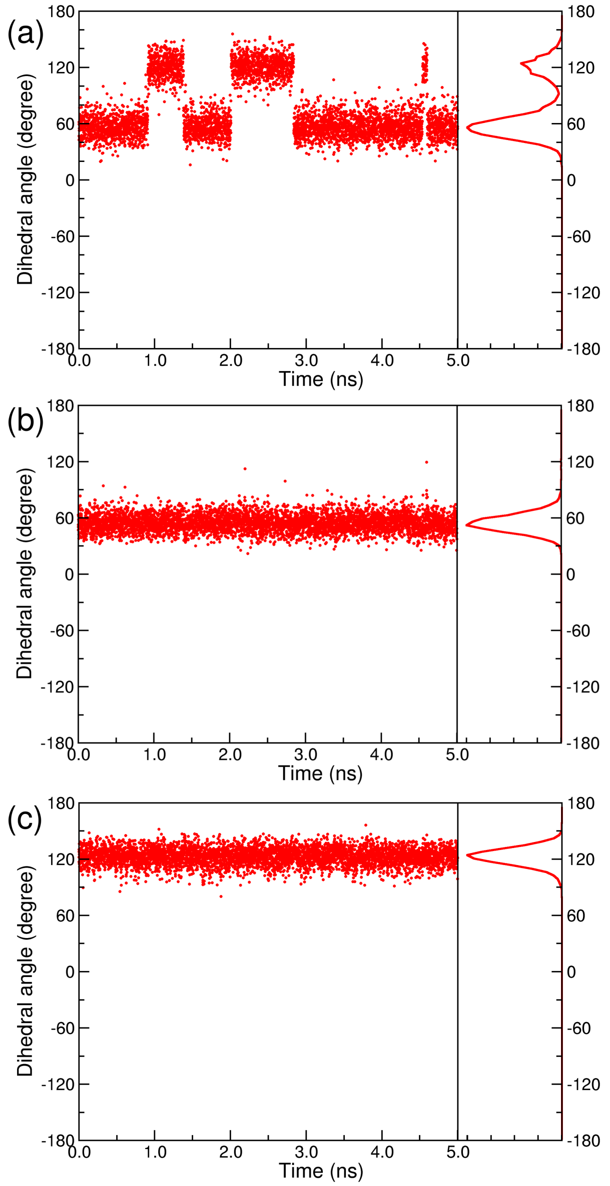

Figure S5: Time dependent dihedral distribution function for dihedral angle **1** for the first 5 ns of simulation in ACN (a), cyclohexane (b), *o*-xylene (c).

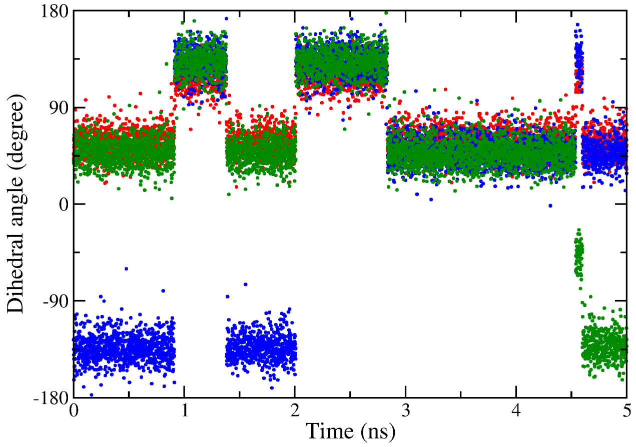

Figure S6: Time dependent dihedral angle distribution function in acetonitrile (in red results for dihedral angle **1**, in blue for dihedral angle **2** and in green for dihedral angle **3**). The first 5 ns of simulation are reported.

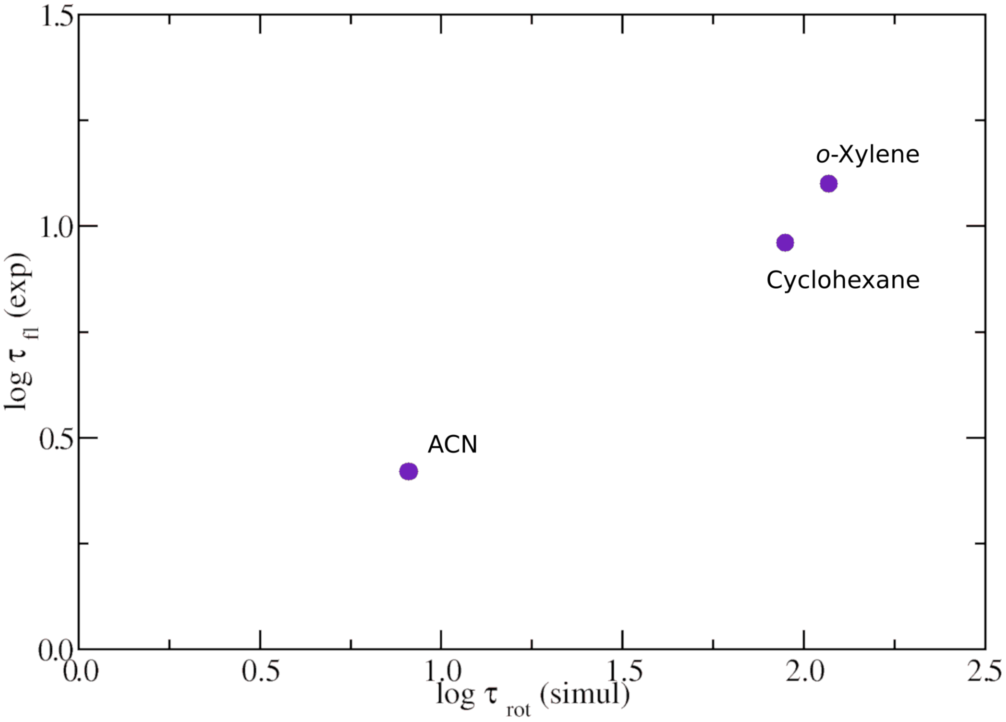

Figure S7: Correlation between DPAP rotational correlation time (ps) and experimental fluorescence lifetimes (ns) in the considered environments.

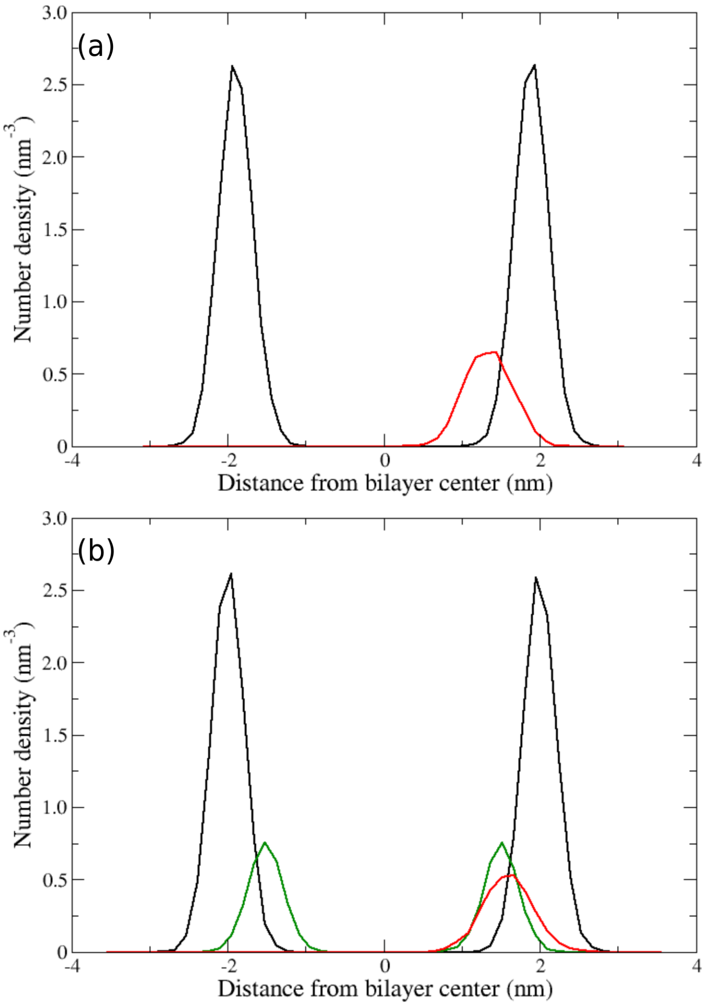

Figure S8: Density profile along the Z-axis for the DPPC/DOPC Phosphorus atoms (black), CHOL Oxygen atoms (green) and DPAP (red). (a) DOPC and (b) DCCP:CHOL membrane bilayers. The number of atoms in the two systems are: 200 P atoms for DOPC, 240 P atoms for DPPC, 75 O atoms for CHOL and 36 for DPAP.

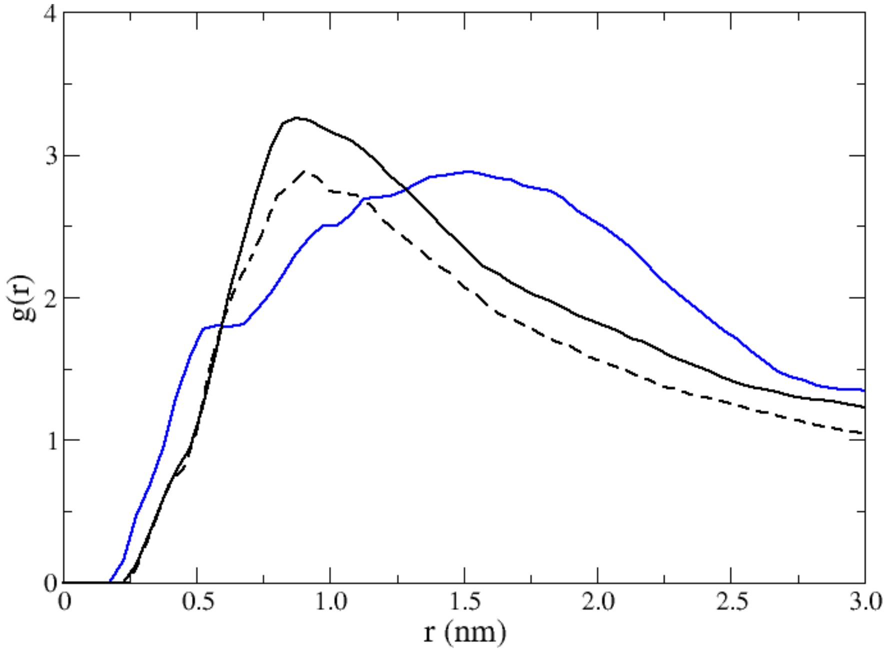

Figure S9: Radial distribution functions between DPAP and DPPC Phosphorus atoms (solid line, black), CHOL Oxygen atoms (solid line, blue), and DOPC Phosphorus atoms (dashed line, black).
